# Organizational Factors Influencing Implementation of Evidence-Based Practices for Integrated Treatment in Behavioral Health Agencies

**DOI:** 10.1155/2014/802983

**Published:** 2014-03-03

**Authors:** Caroline A. Bonham, David Sommerfeld, Cathleen Willging, Gregory A. Aarons

**Affiliations:** ^1^Center for Rural and Community Behavioral Health, Department of Psychiatry, University of New Mexico, MSC09 5030, Albuquerque, NM 87131, USA; ^2^University of California, San Diego, 9500 Gilman Drive (0812), La Jolla, San Diego, CA 92093-0812, USA; ^3^Pacific Institute for Research and Evaluation, Behavioral Health Research Center of the Southwest, 612 Encino Place NE, Albuquerque, NM 87102, USA

## Abstract

*Objective*. In recent years, New Mexico has prioritized integrated treatment for cooccurring mental health and substance use disorders within its public behavioral health system. This report describes factors likely to be important when implementing evidence-based practices (EBPs) in community agencies. *Methods*. Our mixed-method research design consisted of observations, semistructured interviews, and surveys undertaken with employees at 14 agencies at baseline and after 18 months. We developed four-agency typologies based on iterative coding and analysis of observations and interviews. We then examined survey data from employees at the four exemplar agencies to validate qualitative findings. *Results*. Financial resources and strong leadership impacted agency capacity to train providers and implement EBPs. Quantitative analysis of service provider survey responses from these agencies (*N* = 38) supported qualitative findings and demonstrated significant mean score differences in leadership, organizational climate, and attitudes toward EBPs in anticipated directions. *Conclusion*. The availability of strong leadership and financial resources were key components to initial implementation success in this study of community agencies in New Mexico. Reliance only on external funding poses risks for sustainment when demoralizing work climates precipitate employee turnover. Strong agency leadership does not always compensate for deficient financial resources in vulnerable communities.

## 1. Introduction

Despite substantial comorbidity of mental health and substance use disorders [1], integrated treatment is rarely offered in community settings [2]. Since integrated treatment is associated with improved mental health and substance use outcomes [3], as well as reductions in hospitalizations and arrests [4], this failure represents a significant public health concern.

When treating clients with cooccurring disorders, providers must select appropriate clinical interventions [5]. Several specific evidence-based practices (EBPs) fall under the rubric of integrated treatment. EBPs can be defined as “approaches to prevention or treatment that are based in theory and have undergone scientific evaluation” [6]. For example, Dialectical Behavioral Therapy [7] and Seeking Safety [8] have been shown to improve both mental health and substance abuse outcomes in populations with cooccurring disorders. These interventions and similar EBPs help providers deliver comprehensive treatment that addresses both disorders simultaneously. In recent years, research into EBP implementation has become recognized as an important area of study in order to improve health outcomes by identifying how to best translate effective clinical interventions into the US healthcare system [9, 10].

In 2004, the predominantly rural state of New Mexico (NM) prioritized integrated treatment and use of EBPs within the public sector and obtained federal funding to provide training in these modalities. However, implementation of new practices often involves organizational change and is rarely a simple problem that can be addressed with a single series of training sessions [11]. Organizational factors are important predictors of implementation [12, 13]. Agency culture and climate can affect provider attitudes toward adopting EBPs [14]. Positive leadership styles are associated with supportive organizational climates and receptive staff attitudes toward EBPs [15]. Thus, agency leadership is one potential route for modifying organizational climate and encouraging utilization of EBPs [16, 17].

Our study is informed by the Consolidated Framework for Implementation Research in Health Services [18], which suggests that leadership, resources, and access to knowledge and information play crucial roles in determining whether an organization is ready to adopt new practices. Our mixed-method study clarifies how these factors influence delivery of EBPs for integrated treatment in community agencies and identifies agency profiles that can facilitate or hinder these changes.

## 2. Methods

### 2.1. Study Design and Sampling

We employed a mixed-method research design between 2006 and 2008 as part of a long-term study of broader reform efforts in NM and their effects on services for adults with serious mental illness (SMI). This research took place in three rural counties and three counties with metropolitan areas; each was chosen on the basis of geographical diversity and catchment area characteristics (e.g., predominance of ethnic minority versus white residents). We collected qualitative and quantitative data on organizational leadership, climate and finances, training, and provider attitudes toward EBPs and use of these interventions from the 14 behavioral health agencies that provided the majority of services within each county. Although relatively small in size in comparison to agencies located elsewhere in the nation, they reflect the state's range of provider organizations.

Within each agency, we used a purposive sampling approach to identify and recruit participants [19]. We first interviewed a lead administrator, who then referred us to all service providers (e.g., psychiatrists, social workers, counselors, and case managers) and support staff who worked with adults with SMI. Characteristics of participating providers are presented in Table 1.

As shown in Table 2, we conducted semistructured interviews and quantitative surveys with participants at baseline (Time 1) and after 18 months (Time 2). One of the original agencies closed, resulting in a total of 13 agencies at Time 2.

### 2.2. Qualitative Data Collection and Analysis

We developed semistructured interview protocols tailored to administrators, providers, and staff. At each wave of data collection, the research team conducted observations within each agency to better understand contextual factors affecting EBP implementation [20]. When conducting qualitative interviews at Time 2, interviewers used a slightly modified interview protocol and asked about recent agency changes. The research team took extensive field notes and observations which were typed and uploaded to an electronic database. All participants agreed to recorded interviews, which lasted 45–60 minutes, and were professionally transcribed.

The first and third authors coded the interviews and observations using NVivo software [21]. We used a descriptive coding scheme based on questions from the interview protocol and the broader domains of our conceptual model. The resulting codes centered on leadership, training, financial resources, EBP utilization, and workplace morale. When analyzing observation data, we first used open coding to locate themes. We then used focused coding to determine which themes emerged frequently and which represented particular concerns [22]. Discrepancies in coding and analysis were resolved during team meetings through a process of consensus by comparing and contrasting the content of reports, field notes, and interview transcripts [23].

Next, we identified distinct agency profiles associated with the likelihood of implementing EBPs related to integrated treatment. Interventions in the National Registry of Evidence-Based Programs and Practices were identified as EBPs [6]. All references within the interview transcripts to these EBPs were tallied for each agency at both Time 1 and Time 2. Agencies were considered to be in the process of implementing an EBP if multiple providers at Time 2 referred to using the intervention and if observation notes from that agency provided additional confirmation that the EBP was routinely available. We examined all references to EBPs within the interview transcripts and observation notes to identify patterns of potential influences.

Through this analysis, we identified four agencies that best illustrated each of the profiles and exemplified the patterns manifest across the entire dataset. These four agencies served as instrumental case studies providing insight and context into conditions that promoted or hindered EBP implementation [24]. The interviews and observation notes from the remaining agencies were then reexamined to ensure that conclusions from the case studies could be generalized across sites.  Table 3 summarizes agency characteristics.

After analyzing the qualitative data, we hypothesized the following: organizational climate was a function of (1) leadership and (2) access to resources and that (3) providers at agencies with positive work climates would be favorably disposed towards use of EBPs. We then assessed these hypotheses with Time 2 quantitative data from the four agencies serving as case studies.

### 2.3. Quantitative Data and Analysis

For the quantitative investigation, we compared the average ratings of leadership, demoralizing climate, and attitudes toward EBPs from the providers employed by the four agencies (*N* = 38). Missing data regarding attitudes toward EBPs reduced the total number of provider ratings to 34 for this measure. Each measure is described below.

#### 2.3.1. Transformational Leadership

The Multifactor Leadership Questionnaire (MLQ) [25] is a widely used measure of leadership in organizations. The MLQ scores have been associated with organizational climate in behavioral health agencies [14]. For this research, we focused on transformational leadership, comprised of five subscales including idealized influence attributed (four items, *α* = .91), idealized influence behavioral (three items, *α* = .86), inspirational motivation (four items, *α* = .94), intellectual stimulation (four items, *α* = .93), and individual consideration (four items, *α* = .87). We asked participants to indicate the extent to which their supervisor demonstrated each behavior measured by the MLQ. These behaviors were rated on a 5-point scale ranging from 0, “not at all,” to 4, “to a very great extent.”

#### 2.3.2. Demoralizing Climate

Demoralizing climate was measured by items from the Children's Services Survey [26], adapted from studies of diverse workplaces, and used previously to assess climate in public mental health agencies [27]. The subscales measuring demoralizing climate include depersonalization (five items, *α* = .85), emotional exhaustion (six items, *α* = .94), and role conflict (nine items, *α* = .88). As with the MLQ, each statement was rated on the same 5-point scale described above.

#### 2.3.3. Attitudes toward EBPs

The Evidence-Based Practice Attitudes Scale (EBPAS) is a 15-item measure that assesses mental health and social service provider attitudes toward adopting EBPs [28]. EBPAS items are also rated with the same 5-point scale. Cronbach's alpha reliability for the overall EBPAS is good (*α* = .79), with subscale alphas ranging from 0.93 to 0.66 [29]. The EBPAS total scale score (used in the present study) represents participants' global attitudes toward adoption of EBPs.

We conducted ANOVAs to assess differences in mean ratings of transformational leadership, demoralizing climate, and EBPAS scores at the four selected agencies. When significant differences were found, we used post hoc analyses to assess all pair-wise comparisons to identify which specific typologies differed from each other on the measure. We minimized the inflated risk of a Type I error by using the Bonferroni adjustment. To explicitly examine the role of leadership, we also conducted *t*-tests comparing the combined service provider ratings from the two agencies with strong leadership compared to the combined service provider ratings from the two agencies with weaker leadership.

## 3. Qualitative Results

Four distinct agency types were associated with the adoption of EBPs related to integrated treatment within the study period: strongly facilitative, resource driven, leader driven, and resource deprived. The following case studies illustrate how leadership, access to resources, and training opportunities can influence the implementation process.

### 3.1. Strongly Facilitative (Agency A)

“Strongly facilitative” agencies implemented EBPs through supportive leadership and external funding. Agency A was led by a popular administrator, who ensured that providers were trained in EBPs and had time for weekly supervision. These efforts helped to establish a constructive organizational climate where providers described feeling motivated and challenged by their work. By Time 2, providers regularly incorporated five EBPs into clinical work. All data from this agency indicated that the EBPs were part of routine care. During interviews, providers spoke about these interventions knowledgably; the agency's website provided clear summaries of the EBPs; and observation data confirmed that providers had access to necessary materials. This agency participated in the National Institute on Drug Abuse Clinical Trials Network. Through this network, agency personnel identified funding opportunities and successfully competed for external grants that covered the high cost of training and buffered the agency from lost income when providers did not generate revenue through the provision of direct services.

One provider underscored the importance of a positive organizational climate when working in a field with many clinical challenges.

 
*“I like the staff I work with, I wouldn't be working here if it wasn't that way…There's a lot of laughter, and that means a lot to me…The stressor is the nature of the work…Working with mental illness, and drug and alcohol clients—it's draining. You're dealing with crises a lot.”*

Unlike personnel in other agencies, all providers commented on the high workplace morale; the majority also felt prepared to deliver integrated treatment.

### 3.2. Leader Driven (Agency B)

At “leader driven” agencies, strong managers initiated EBPs despite limited funding. Agency B served an economically challenged rural region with high rates of substance use. The clinic had minimal infrastructure and considerable financial stress. When interviewed at Time 1, providers were largely unfamiliar with EBPs and had difficulty naming any interventions based on research. Eighteen months later, the agency was consistently offering one new EBP. However, because of financial constraints, none of the providers received formal training and they expressed doubts about their abilities to deliver this EBP effectively.

 
*“It would've been nice to have some training…They had talked about training, but then they just threw us in there. We reviewed (the manual) and just started piecing it together, and we probably didn't do it right at first.”*

Just prior to the launch of this new EBP, the agency hired a popular clinical director who prioritized education and established regular supervision sessions. In response to provider requests for more education, he instituted regular supervision sessions. However, he was worried that these sessions were insufficient when teaching new material.

 
*“Folks need help, support, and training. They need more time with their supervisor. The supervisor needs more training and more help. It's just that if it's not billable, we can't do it. That's what it boils down to…(we) can't bill for training, can't get reimbursed for training.”*

When first learning interventions, both providers and supervisors needed opportunities to ensure that their knowledge of the practice was complete. Without time, funding, and educational resources, it was difficult to implement EBPs with fidelity.

Despite receiving personal support from the director, providers at this agency felt overwhelmed. Ultimately there was high turnover. Providers described a lack of community resources that hampered appropriate treatment for people with cooccurring disorders. When asked why work was stressful, they emphasized daunting community needs rather than specific complaints about internal agency management.

### 3.3. Resource Driven (Agency C)

Resource driven agencies relied primarily on external funding and workshops to implement new practices. Agency C served a rural community and operated several clinic sites. After learning that EBPs were eligible for higher reimbursement rates, the lead administrator hired staff to pursue grant opportunities to diversify the organization's funding portfolio. These resources allowed the agency to send providers to trainings. As in the case of the leader driven agency, few had familiarity with EBPs at Time 1. However, by Time 2, many clinicians had adopted up to four EBPs. They spoke positively about these changes, asserting that EBPs provided structure to an otherwise chaotic work environment.

Time constraints impeded the dissemination process. Although group supervision sessions were potentially available, most providers could not attend them regularly because of clinical commitments and the logistics of traveling between offices.

At this agency, providers routinely expressed frustration with their work environment. These frustrations reflected the challenges of rural practice: working in small teams, staffing several offices, and providing services to clients with complex needs over a large geographical area.

The relatively rapid uptake of several practices confirms that access to additional financial resources facilitated implementation. Nevertheless, a number of providers confided that they were thinking of leaving the agency, which would disrupt efforts to sustain these changes.

### 3.4. Resource Deprived (Agency D)

In “resource deprived” settings, weak leadership and inadequate funding discouraged the use of EBPs. Our fourth case study involved a rural agency with a history of collaborating with university researchers on the adaptation of existing EBPs for different cultural populations. During Time 1, providers were enthusiastic about EBPs and spoke positively about their relationship with the university. However, by Time 2, the research relationships were in transition as agency leadership changed. Previous grants were ending and program funding was unlikely to be renewed. At this time, providers were more skeptical about the value of EBPs. They also felt disconnected from the world of academia and research. One provider explained the following.

 
*“Evidence-based practices show significance, but I don't see the benefits at a local level…(Researchers) come in and use their evidence-based approaches…With the information they collect, they take it back…I don't see what in the world they do with it.”*

Finally, in the climate of fiscal stress and leadership changes, many providers expressed low morale. Due to financial problems, the agency lost several employees. Some found better paying jobs; the contracts of others were not renewed when grants ended. Although at Time 1 several providers used EBPs regularly, by Time 2, none of the providers were using these interventions. Some said it was just not possible to deliver EBPs without sufficient staff. In this setting, turnover not only of providers but also among leadership emerged as barriers to implementation efforts.

## 4. Quantitative Results

Based on our qualitative analysis, we had anticipated that strongly facilitative and leader driven agencies would have higher scores for transformational leadership than the resource driven and resource deprived agencies. We additionally expected that the strongly facilitative agency would have the lowest demoralizing climate and that the resource deprived agency would have the highest. Finally, we anticipated that the relationship between agency leadership and resources would be evident in provider attitudes toward EBPs. We also predicted that providers at the strongly facilitative agency would report more positive experiences with EBPs whereas the providers at the resource deprived agency might be more skeptical. As shown in Table 4, survey results indicated that ratings of agency leadership and organizational climate were generally consistent with the qualitative findings. The ANOVA test for transformational leadership did not identify significant differences between the four agencies, likely due to the small sample size within agency. However, as anticipated, the mean scores for the two agencies with stronger leadership were higher than for the two agencies with poor leadership. Significant differences emerged when we grouped service providers from the strongly facilitative and leader driven agencies and compared them to their counterparts at the resource driven and resource deprived agencies (*t* = 2.06, *P* = 0.046).

The results for demoralizing climate partially met expectations since we found significant differences in scores across the four agencies (*F* = 3.91, *P* = 0.02). While the strongly facilitative agency had the lowest demoralizing climate score, post hoc analysis indicated that the primary difference was between the strongly facilitative and leader driven agencies (*P* = 0.024).

Quantitative analysis of service provider attitudes toward EBPs also supported the patterns found in the qualitative results. We found significant differences between the four agencies (*F* = 5.03, *P* = 0.01). Post hoc analysis using the Bonferroni correction indicated that service providers at the strongly facilitative agency had more favorable attitudes toward EBPs than providers from the resource deprived agency (*P* = 0.003).

## 5. Discussion

According to the Consolidated Framework for Implementation Research, the three major components that predict an organization's readiness for implementation are leadership engagement, availability of resources, and access to information and knowledge. We used multiple methods to examine how these three factors shaped organizational climate and prepared providers for the implementation of new practices in publicly funded agencies in NM. Our mixed-method approach used quantitative data to examine and validate our qualitatively derived organizational typology. In keeping with previous studies [14, 30, 31] and our qualitative findings, the survey results suggested that the presence of strong leadership and adequate financial resources affect provider attitudes toward EBPs and facilitate implementation of these innovations.

There were consistent patterns in qualitative data linking use of EBPs with leadership and organizational climate across the 14 participating agencies. In agencies that had implemented multiple EBPs by Time 2, providers frequently described a sense of camaraderie at work which they generally attributed to the presence of supportive leaders.

The higher transformational leadership scores at the strongly facilitative and the leader driven agencies corresponded with qualitative descriptions of supportive leaders who prioritized individual supervision, were available when questions arose, and made efforts to praise and reward employees for their work. These descriptions are consistent with the construct of transformational leadership which includes close supervisory relationships and is exemplified by leaders who make efforts to meet the needs of supervisees through individualized feedback, support, and motivation [32, 33]. Transformational leadership has previously been associated with positive attitudes toward EBPs [15] and organizational support for EBP [31]; our study emphasizes its particular importance during active EBP implementation.

Our findings regarding the association between transformational leadership and demoralizing climate suggest that this relationship is indirect. In our study, the leader driven agency provided an example of strong leadership amidst a demoralizing work climate. The qualitative data from this site indicated that the consistently low ratings of organizational climate were in response to the pervasive poverty of the region rather than reflecting agency leadership. And, although we had hypothesized that the resource deprived agency would have the most demoralizing climate, it appeared that lacking either leadership or financial resources was similarly detrimental to organizational climate. In chronically underfunded settings with considerable unmet need, an overemphasis on leadership may divert attention from the enduring problem of scarce resources. Interestingly, other studies of EBP implementation in substance use treatment settings have noted that the lack of financial resources is a major contributor to stress among front line staff yet can go unrecognized as a barrier by agency leadership [34].

Our third hypothesis was that the readiness of an organization to implement new EBPs would reflect a combination of agency leadership, access to resources, and training opportunities as exemplified by the strongly facilitative agency. Indeed, this agency implemented the most EBPs and demonstrated the highest scores on the EBPAS scale by Time 2. The strongly facilitative agency also experienced the least provider turnover during the study period which corresponded with the positive organizational climate and also facilitated the uptake of new EBPs.

Through the qualitative data collected at all sites, we observed a bidirectional relationship between organizational climate and provider turnover. Poor organizational climates tended to precipitate turnover. Recent turnover and a lack of a consistent workforce likewise contributed to a stressful work climate. The agencies in this study experienced substantial changes in staffing over the course of 18 months. This high level of turnover is not uncommon in community agencies [26, 35, 36]. Further analysis confirmed that turnover was precipitated by negative organizational climate but was somewhat ameliorated by strong leadership [4]. When implementing new EBPs, ongoing staff turnover may be especially problematic if agencies rely primarily upon a “resource driven” approach requiring substantial investments in introductory training sessions. While such trainings can facilitate initial implementation, they are unlikely to encourage sustainment of new practices in demoralizing climates with high turnover.

The experience at the leader driven agency suggests that a supportive supervisor can spearhead implementation initiatives. However, the agency's limited capacity meant that only a single EBP was initiated, whereas, agencies with higher levels of external funding were able to implement multiple EBPs during the eighteen-month timeframe. Additionally, the agency's inability to send providers to training sessions raises concerns about whether EBPs can be delivered with fidelity in settings under such financial strain. This experience mirrors previous research findings that agency prioritization and trainings are initial facilitators of EBP implementation, but that adequate funding is essential for long term sustainment [30, 37]. Additionally, other work supports the premise that efforts to implement EBPs without adequate resources are associated with increased likelihood of adaptations and ultimately decreased fidelity [38]. When planning implementation strategies in underresourced rural areas in particular, capacity building is paramount. Initial costs can be considerable when carrying out new practices [39] and periods of reduced revenue are common during startup phases [40, 41].

Other considerations when implementing EBPs include the relationships between agencies and researchers. Community-based participatory research is recognized as a facilitator for the adoption of science-based interventions in community settings [42, 43]. The experience at the strongly facilitative agency supports this model. Proactive leadership promoted collaboration with researchers which led to grant funding and strengthened implementation efforts. Additionally, we observed that clinicians with personal experience of research were more positively disposed toward EBPs, a finding replicated in other settings [44]. However, principles of community-based participatory research emphasize long-term relationships between community members and academics [45]. While such a partnership had met with success in the resource deprived agency, this relationship was not sustained when organizational leadership changed. Subsequently, when providers were reinterviewed at Time 2, they expressed distrust about the research process. This distrust appeared to correspond with their diminished use of EBPs. Leadership turnover has also been identified as a critical factor in sustainment in an 8-year longitudinal study of EBP implementation [46].

States have an influential role in the implementation of EBPs in public settings [47, 48], and ongoing state funding and state sponsored training are associated with long term sustainment [49]. The relatively rapid increase in EBP provision in agency settings in NM reflects policy changes and investment in initial training made at the state level. However, this process is incomplete and we do not know the degree to which EBPs were implemented with fidelity. Integrated treatment can be difficult to implement with high fidelity [50]. In this study, we did not assess fidelity outcomes but ethnographic findings published elsewhere suggest that fidelity and sustainment will be long-term concerns across the state [51].

Strengths of our study include its longitudinal design and focus on community agencies. However, the observational design of this study and the small sample of agencies and providers limit the generalizability of our findings. We focused on publicly funded agencies serving adults with serious mental illness and cooccurring substance use disorders and did not assess the experiences of independent practitioners and primary care providers who deliver limited behavioral health services or providers specializing in treating youth. Finally, this research may not fully generalize to other states.

## 6. Conclusions

In this study, agency leadership and the availability of external financial resources influenced EBP uptake. These factors facilitated provider education, a necessary step for changing clinical practice. While the funding facilitated initial training sessions, consistent leadership nurtured supportive work climates, minimized provider turnover, and encouraged sustainment. Clinical managers encouraged new practices through regular supervision sessions. Yet, strong agency leadership did not always compensate for the lack of resources and existing infrastructure. Future reform should consider these disparities when planning fiscal resources for vulnerable communities.

## Figures and Tables

**Table 1:** Demographics of sample.

	%
Gender	
Male	28
Female	72
Position at agency	
Clinician	56
Support staff	24
Upper level administrator	20
Education	
<8th grade	0.5
Completed high school	11
Some college	30
Completed college	20
Some graduate education	4
Completed master's degree	28
Completed doctorate (PhD, MD, and/or Ed.D)	6
Missing/other	5
Ethnicity	
Nonhispanic White	44
Hispanic	39
Native American	17
Other	3
Mean age of participants	46 years

**Table 2:** Sequence of data collection.

Time 1 (baseline)		Time 2 (18 months later)	
14 agencies		13 agencies	
Qualitative data		Qualitative data	
Observations		Observations	
24 hours at each agency		At least 8 hours at each agency	
Semistructured interviews		Semistructured interviews	
Direct service providers	(*n* = 110)	Direct service providers	(*n* = 93)
Support staff	(*n* = 41)	Support staff	(*n* = 30)
Upper level administrators	(*n* = 39)	Upper level administrators	(*n* = 27)
		Quantitative data	
		Attitudes towards evidence-based practices	(*n* = 34)
		Transformational leadership	(*n* = 38)
		Demoralizing climate	(*n* = 38)

**Table 3:** Characteristics of agencies.

Agency	Rural or urban setting	Agency size^a^	Degree of staff turnover^b^	Number of EBPs at Time 1	Number of EBPs at Time 2
Agency increased uptake of EBPs					
*Serves homeless population	**Urban**	**Medium**	**Low**	**3**	**5**
**Community mental health center	**Rural**	**Medium**	**High**	**0**	**1**
***Community mental health center	**Rural**	**Large**	**Moderate**	**2**	**4**
Substance abuse treatment facility	Urban	Medium	Moderate	1	3
Community mental health center	Urban	Medium	Moderate	0	2
Community mental health center	Rural	Large	Moderate	0	1
Community mental health center	Rural	Small	High	2	3
Agency decreased use of EBPs					
****Substance abuse treatment facility	**Rural**	**Large**	**Moderate**	**2**	**0**
Community mental health center	Urban	Large	Moderate	1	0
Small group practice	Rural	Small	N/A	2	Closed
Agency had no change in use of EBPs					
Community mental health center	Urban	Large	Low	2	2
Substance abuse treatment facility	Rural	Large	Moderate	2	2
Small group practice	Urban	Small	High	0	0
Serves homeless population	Urban	Small	Low	0	0

*Agency A: strongly facilitative.

**Agency B: leader driven.

***Agency C: resource driven.

****Agency D: resource deprived.

^a^Small agency: less than 8 employees; medium agency: 9–14 employees; large agency: 15–21 employees.

^b^Low turnover: less than 25%; moderate turnover: between 25–50%; high turnover: more than 50%.

**Table 4:** Service provider ratings of transformational leadership, demoralizing climate, and EBP attitude by agency.

Agency	*N *	Mean	S.D.	S.E.	*F *	Sig.
Transformational leadership	10	2.55	0.69	0.22		
A—strongly facilitative	5	2.62	1.58	0.71	1.36	0.27
B—leader driven	15	1.87	1.15	0.3		
C—resource driven	8	1.78	1.11	0.39		
D—resource deprived						
Demoralizing climate						
A—strongly facilitative	10	0.61	0.39	0.12		
B—leader driven	5	1.69	0.5	0.22	3.91	**0.02**
C—resource driven	15	1.21	0.63	0.16		
D—resource deprived	8	1.36	0.92	0.33		
Attitudes towards EBPs						
A—strongly facilitative	8	3.08	0.41	0.14		
B—leader driven	5	2.67	0.46	0.21	5.03	**0.01**
C—resource driven	13	2.61	0.45	0.13		
D—resource deprived	8	2.2	0.5	0.18		
